# Pterygium Screening and Lesion Area Segmentation Based on Deep Learning

**DOI:** 10.1155/2022/3942110

**Published:** 2022-11-21

**Authors:** Shaojun Zhu, Xinwen Fang, Yong Qian, Kai He, Maonian Wu, Bo Zheng, Junyang Song

**Affiliations:** ^1^School of Information Engineering, Huzhou University, Huzhou 313000, China; ^2^Zhejiang Province Key Laboratory of Smart Management & Application of Modern Agricultural Resources, Huzhou University, Huzhou 313000, China; ^3^Jiangsu Testing and Inspection Institute for Medical Devices, Nanjing 210000, China; ^4^Department of Ophthalmology, The First People's Hospital of Huzhou, Huzhou 313000, China

## Abstract

A two-category model and a segmentation model of pterygium were proposed to assist ophthalmologists in establishing the diagnosis of ophthalmic diseases. A total of 367 normal anterior segment images and 367 pterygium anterior segment images were collected at the Affiliated Eye Hospital of Nanjing Medical University. AlexNet, VGG16, ResNet18, and ResNet50 models were used to train the two-category pterygium models. A total of 150 normal and 150 pterygium anterior segment images were used to test the models, and the results were compared. The main evaluation indicators, including sensitivity, specificity, area under the curve, kappa value, and receiver operator characteristic curves of the four models, were compared. Simultaneously, 367 pterygium anterior segment images were used to train two improved pterygium segmentation models based on PSPNet. A total of 150 pterygium images were used to test the models, and the results were compared with those of the other four segmentation models. The main evaluation indicators included mean intersection over union (MIOU), IOU, mean average precision (MPA), and PA. Among the two-category models of pterygium, the best diagnostic result was obtained using the VGG16 model. The diagnostic accuracy, kappa value, diagnostic sensitivity of pterygium, diagnostic specificity of pterygium, and F1-score were 99%, 98%, 98.67%, 99.33%, and 99%, respectively. Among the pterygium segmentation models, the double phase-fusion PSPNet model had the best results, with MIOU, IOU, MPA, and PA of 86.57%, 78.1%, 92.3%, and 86.96%, respectively. This study designed a pterygium two-category model and a pterygium segmentation model for the images of the normal anterior and pterygium anterior segments, which could help patients self-screen easily and assist ophthalmologists in establishing the diagnosis of ophthalmic diseases and marking the actual scope of surgery.

## 1. Introduction

Pterygium is a common and frequently occurring disease in ophthalmology that affects the fibrovascular tissue on the ocular surface, resulting in eye irritation and inflammation [1, 2]. It can cause visual impairment or even blindness when the lesion covers most of the cornea [3, 4]. Corresponding treatment methods can be used to control pterygium development in the early stage. However, in the later stage, only surgery can be used to respect the lesion area for treatment [5–7]. The diagnosis and surgery of pterygium require the localization of the lesion area. Currently, the most commonly used method is manual positioning by ophthalmologists based on anterior segment images. Manual positioning is slow and not precise, and different doctors may position different lesion ranges. Simultaneously, the early detection, diagnosis, and treatment of pterygium can better control or treat the disease. Therefore, a pterygium two-category model and a pterygium lesion area segmentation model were designed, which could initially screen the pterygium and segment the lesion area accurately. These models can assist ophthalmologists in establishing the diagnosis of ophthalmic diseases and marking the scope of surgical resection.

With the close integration of artificial intelligence and ophthalmology, many studies have used deep learning models to assist in the diagnosis of ophthalmic diseases [8–13]. In terms of lesion segmentation, most studies have diagnosed glaucoma by segmenting the optic disc [14–16], and there have also been some studies on segmenting the blood vessels of fundus images to screen for related diseases [17–19]. Regarding the studies conducted on pterygium, some researchers used traditional machine learning [20] and deep learning methods to classify [21, 22] pterygium as normal and pterygium disease. A three-category pterygium model on normal, pterygium observation, and pterygium surgery periods was studied by some researchers [23]. Related studies have also been conducted on the localization and segmentation of pterygium lesions [24]. The above studies on pterygium classification and segmentation were conducted separately. In this study, the two studies were combined. The two-category model of pterygium was used on the anterior segment image, and the lesion area was segmented according to the pterygium image.

In this study, four deep-learning models were used to realize the two categories of pterygium for preliminary screening. Simultaneously, the team's improved models were used to segment the pterygium lesion area accurately, which could not only help patients understand the progression of pterygium but could also assist ophthalmologists in establishing the diagnosis of ophthalmic diseases and marking accurate lesion localization before surgery.

## 2. Materials and Methods

### 2.1. Data Source

The Affiliated Eye Hospital of Nanjing Medical University provided 1034 anterior segment images for this study. The data were obtained using two different brands of slit-lamp digital microscopes, and the quality of the images was high. Relevant personal information of the patient was removed from the image data provided. Therefore, it did not violate the patient's privacy. This study had no restrictions on the sex and age of patients, and the data provided did not contain related information of patients. Hence, this study had no relevant statistics.

**Figure 1:**
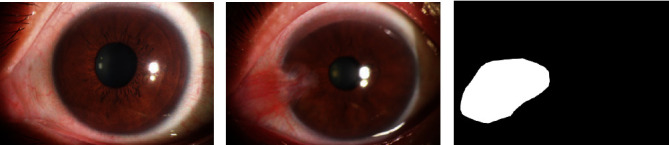
Images of normal anterior segment, pterygium anterior segment, and the labeling map of the lesion area.

The anterior segment images provided by the hospital in this study were of a single type of pterygium, which can only be diagnosed as normal or pterygium. The corresponding label (normal or pterygium) of each anterior segment image and lesion area annotation map of the pterygium anterior segment image along with the image were provided by the hospital. The marking standard for pterygium was as follows [25]: the normal anterior segment was characterized by the absence of evident hyperemia or proliferative bulge in the conjunctiva, with a transparent cornea.  Figure 1 shows the images of the normal anterior segment Figure 1(a), the anterior segment of the pterygium Figure 1(b), and the labeling map of the lesion area Figure 1(c). Two professional ophthalmologists independently diagnosed the same anterior segment. If the diagnosis results were consistent, it was the final diagnosis result. If the diagnosis results were inconsistent, the final diagnosis result was decided by an expert ophthalmologist. Labeling of the pterygium lesion area was performed by a trained professional ophthalmologist and confirmed by an expert ophthalmologist. If the lesion area was marked incorrectly, it was revised and reconfirmed until it was correct.

The pterygium two-category models were trained using 734 anterior segment images and were tested using 300 anterior segment images. The normal anterior segment and pterygium images in the training and test image data were equally divided. The pterygium lesion area segmentation models were trained using 367 pterygium images and tested using 150 pterygium images.

### 2.2. Classification Model Training

**Figure 2:**
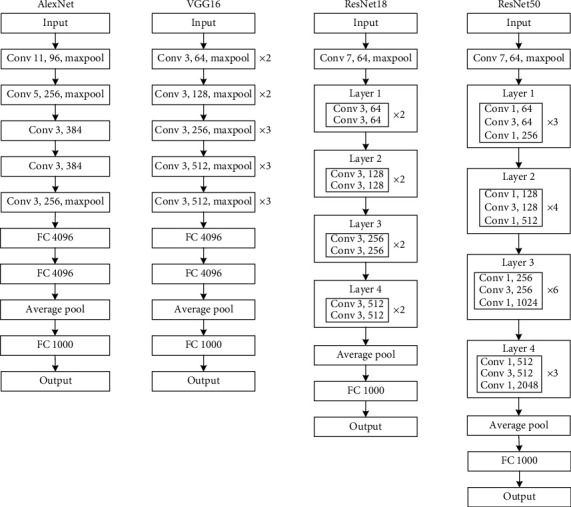
The model network structures of AlexNet, VGG16, and ResNet.

Deep learning classical classification models mainly include AlexNet [26], VGG16 [27], ResNet18 [28], and ResNet50 [28]. This study used the above four classical models to design two-category models on normal and anterior pterygium segment images. The network structures of these classical models are similar. The backbone networks of AlexNet and VGG16 include convolutional, pooling, and fully connected layers. ResNet adds a residual network structure. The model network structure is shown in Figure 2.

The aforementioned classical models require an input image size of 224 × 224 pixels. In this study, the adaptive average pooling method was added before the fully connected layer of the classical models. Therefore, the input size could be adjusted to the required size. The input image size was set to 336 × 224 pixels to adapt to the size of the original anterior segment image.

Normal and pterygium anterior segment images were divided into the training and validation sets in a 9 : 1 ratio. When training the pterygium two-category model, the original image was resized to 336 × 224. The preprocessing method adopted a random rotation of −3° − 3°. The parameters trained by several models in the ImageNet [29] dataset were used as the initial parameters for the corresponding models. The loss function was the cross-entropy loss function. The learning rate of AlexNet and VGG16 was 0.001, the epoch was 30, the learning rate of ResNet18 and ResNet50 was 0.01, and the epoch was 100. The training parameters of the four models were iteratively updated to obtain the best model for the validation set as the final pterygium two-category model for each model.

### 2.3. Segmentation Model Training

Classical semantic segmentation models include U-Net [30], DeepLabv3+ [31], and PSPNet [32] models. The PSPNet and its improved models were used to segment the pterygium lesion areas in the anterior segment images of the pterygium. The results were compared with those of other segmentation models.

**Figure 3:**
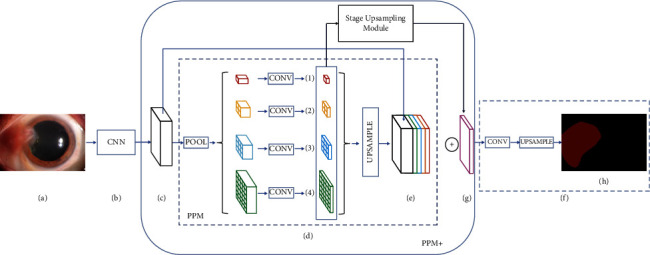
The structures of PSPNet and phase-fusion PSPNet. (a) represents the input image; (b) represents the feature extraction network, the feature extraction part of MobileNet or ResNet50; (c) represents the feature map extracted by the feature extraction network; (d) represents the pyramid pooling module; (e) represents the feature map output by the pyramid pooling module; (f) represents the output module; (g) represents the feature map formed by stage upsampling module; (h) represents the output image.

MobileNet [33] was used as the backbone network of PSPNet to extract features and obtain the feature map of the input image. Average pooling was used on the feature map at four different scales: 1 × 1, 2 × 2, 3 × 3, and 6 × 6. Subsequently, the maps after average pooling with the same size as the feature map were obtained through bilinear interpolation. The feature map and maps after average pooling were spliced; finally, the segmented prediction map was obtained. As shown in Figure 3, PSPNet consists of Figures 3(a)–3(d) and 3(f), excluding Figure 3(g) and the stage upsampling module in PPM+.

The backbone network MobileNet was replaced by ResNet50 in the PSPNet, which can obtain better mean intersection over union (MIOU) and IOU results. Two improvements were made to the PSPNet model using ResNet50 as the backbone network. The first improvement was to increase the stage upsampling module, which first upsampled the feature map (1) to ×2 through bilinear interpolation and then added the sampled feature map and feature map (2). The added feature map was upsampled and then added to the feature map (3) element by element. The added feature map was upsampled and then added to the feature map (4) element by element. The final added feature map was upsampled to 30 × 30 pixels. The feature map obtained after the stage upsampling module continued to be stacked to Figure 3(e) to obtain a new feature map. Therefore, a new pyramid pooling module (PPM+) was obtained, and the final prediction map through convolution was obtained. The first improvement model, called phase-fusion PSPNet, and the structure of this model are shown in Figure 3.

**Figure 4:**
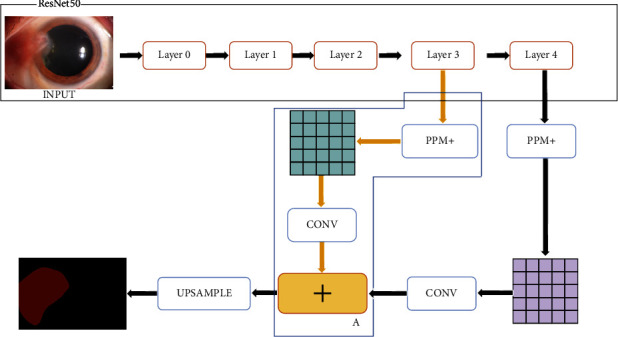
The structure of the double phase-fusion PSPNet.

The second improvement was mainly aimed at the feature extraction of the ResNet50 network. The shallow feature maps of the ResNet50 third-layer input were input into the PPM + module, and the results obtained after convolution were the same as those obtained after PPM+ and convolution in the phase-fusion PSPNet. Feature maps were added, and the final prediction map was obtained after upsampling. As shown in Figure 4, box A in the figure represents the newly added feature extraction and fusion module in the phase-fusion PSPNet.

A total of 367 pterygium anterior segment images were selected to train the segmentation models, of which 330 and 37 were used as the training and validation sets, respectively. Both sides of the short side of the input image were lengthened so that the length of the short side was the same as the length of the long side. Then, the image became a square, and the increased part was filled with gray (*R*, *G*, *B* are all 128), and the square image size was resized to 473 × 473 as the input image for training. The number of training epochs was 80, and the model with the best validation result was selected as the final segmentation model.

### 2.4. Statistical Analyses

The Statistical Package for the Social Sciences version 22.0 software was used for statistical analyses of the two-category models. The count data are expressed as the number and percentage of images. The sensitivity, specificity, F1-score, area under the curve (AUC), kappa value, and other indicators were used to evaluate the diagnosis results of the expert diagnosis and model groups. A receiver operating characteristic (ROC) curve was drawn to compare the results of the models. Segmentation of pterygium lesions was evaluated using four indicators: IOU, MIOU, PA, and MPA.

### 2.5. Calculation Methods

The calculation methods of IOU, MIOU, PA, and MPA are as follows:

(1) $$\begin{array}{c} \begin{array}{c} \text{IOU}=\frac{p_{i}\cap g_{i}}{p_{i}\cup g_{i}} \end{array},\\ \begin{array}{c} \text{MIOU}=\frac{1}{k+1}\sum_{i=0}^{k}\frac{p_{i}\cap g_{i}}{p_{i}\cup g_{i}} \end{array},\\ \begin{array}{c} \text{PA}=\frac{\sum_{i=0}^{k}p_{ii}}{\sum_{i=0}^{k}\sum_{j=0}^{k}p_{ij}} \end{array},\\ \begin{array}{c} \text{MPA}=\frac{1}{k+1}\overset{k}{\underset{i=0}{\sum }}\frac{p_{ii}}{\sum_{j=0}^{k}p_{ij}}, \end{array} \end{array}$$

where *p*_*i*_ is the segmented area, *g*_*i*_ is the real area, *k* is the number of classes (excluding background classes), *p*_*ii*_ is the number of correctly predicted pixels, and *p*_*ij*_ and *p*_*ji*_ are the numbers of incorrectly predicted pixels.

## 3. Results

### 3.1. Results of Classification

**Figure 5:**
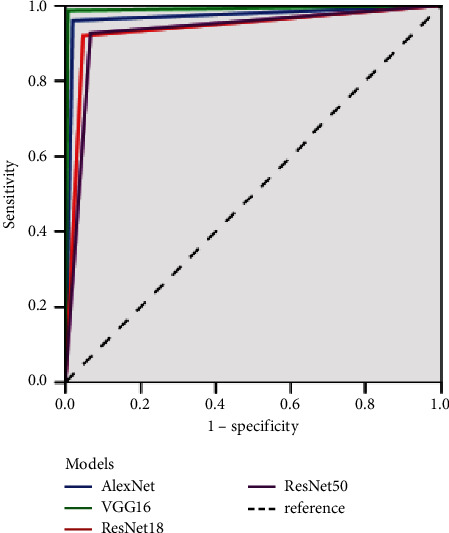
Receiver operating characteristic curve of the four models.

In this study, four models were tested with 150 images of normal and pterygium anterior segments, and the VGG16 model had the best results, with an accuracy of 99% and a kappa value of 98%. The sensitivities of diagnosing normal and pterygium were 99.33% and 98.67%, respectively, the specificities were 98.67% and 99.33%, and the AUCs were 98.67% and 99.33%, respectively. The diagnostic results and evaluation indicators of the four models are shown in Tables 1 and 2, respectively, and the ROC curve is shown in Figure 5.

### 3.2. Results of Segmentation Models

A total of 150 pterygium anterior segment images were used to test U-Net, DeepLabv3+, PSPNet (based on MobileNet and ResNet50), and the two improved models based on PSPNet. The pterygium segmentation results for the six models are presented in Table 3.

**Figure 6:**
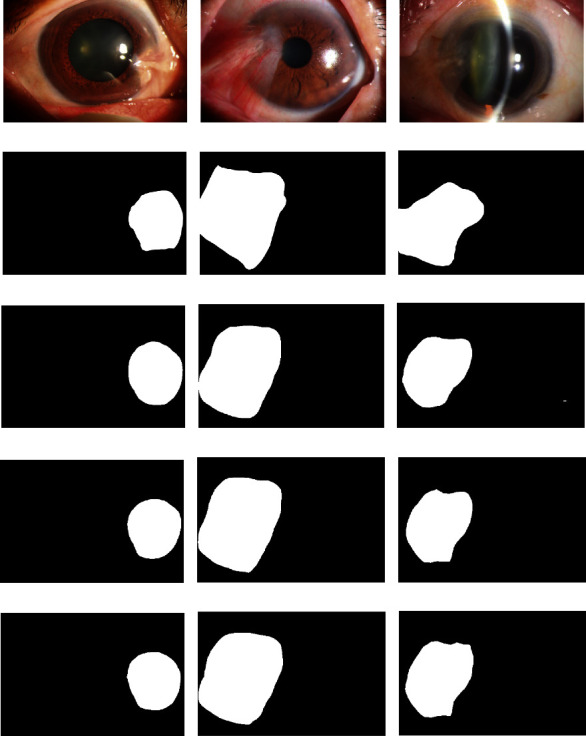
The segmentation results of the phase-fusion PSPNet and double phase-fusion PSPNet. Figures (a)–(c) show the original pterygium images; figures (d)–(f) show the real label of the pterygium lesion area of figures (a)–(c); figures (g)–(i) show the segmentation results of the PSPNet (ResNet50) model; figures (j)–(l) show the segmentation results of the phase-fusion PSPNet model; figures (m)–(o) show the segmentation results of the double phase-fusion PSPNet model.

As shown in Table 3, the PSPNet model based on ResNet50 performed better than the U-Net, DeepLabv3+, and MobileNet-based PSPNet models for the MIOU, IOU, and MPA indicators. The double phase-fusion PSPNet was obtained after two improvements on the ResNet50-based PSPNet; its MIOU, IOU, MPA, and PA were 86.57%, 78.1%, 92.3%, and 86.96%, respectively. The result of the PA was slightly worse than that of the PSPNet model based on MobileNet, but other indicators yielded the best results. The segmentation results of the phase-fusion and double phase-fusion PSPNets are shown in Figure 6.

## 4. Discussion

Most patients with pterygium are outdoor workers, such as fishermen and farmers [34]. In the early stage of the disease, there will be no significant effect on the patient, and the symptoms are similar to ordinary inflammation, which will not attract the attention of the patient. Thus, the disease gradually develops to the stage where surgical treatment is necessary. The pterygium two-category and lesion segmentation model can help patients screen for the disease by themselves and pay attention to the progress of the lesion area. Therefore, the patient has an intuitive understanding of the disease's progress and then immediately visits a hospital for diagnosis and treatment, finally obtaining a good therapeutic effect.

Four classical classification models were selected to diagnose whether the anterior segment images were normal or pterygium images. The normal anterior segment was clearly distinguished from the anterior segment of the pterygium. Subsequently, the features can be extracted better without a complex network structure. Therefore, the VGG16 model yielded the best results. ResNet18 and ResNet50 have more complex network structures, whereas the AlexNet network structure is slightly simpler; therefore, the diagnosis results of these models were both worse than those of VGG16.

In 2018, Wan Zaki et al. [20] used support vector machine (SVM) and artificial neural network methods to study the two categories of pterygium. The data used in the study were obtained from four datasets, including 2692 and 325 images of the normal anterior and pterygium anterior segments, respectively. The result obtained using the SVM method was better, with sensitivity, specificity, and AUC values of 88.7%, 88.3%, and 0.956, respectively. In 2019, Zulkifley et al. [21] used the convolutional neural network method to diagnose pterygium based on 60 normal and anterior pterygium segment images, with diagnostic sensitivity and specificity of 95% and 98.3%, respectively. In this study, the sensitivity, specificity, and AUC of the VGG16 model for the diagnosis of pterygium were 98.67%, 99.33%, and 0.99, respectively, which are higher than those reported by other researchers. The VGG16 model can better extract image features. The training data were balanced, and the number of training images was greater than that in the literature [21]; thus, better results were obtained.

Classical (U-Net, DeepLabv3, PSPNet) and improved models based on PSPNet (phase-fusion PSPNet and double phase-fusion PSPNet) were used to segment pterygium. According to Table 3, the improved model had better segmentation results. The improved model extracted more features from the pterygium image, which can fully combine local features, global features, and features at different levels in the feature extraction network. Their structures can lose less feature information and obtain better segmentation results.

Abdani et al. [24] used Dense Deeplabv2 to segment pterygium in 2020. Compared with the Deeplabv1, Dense Deeplabv1, and Deeplabv2 models, the best MIOU result was 83.81%. The same team designed Group-PPM-Net to segment pterygium in 2021, and the best MIOU result was 86.32% [35]. Cai et al. [36] used DRUNet and SegNet to segment pterygium, and the best IOU was 60.8%. The MIOU and IOU results obtained using the double phase-fusion PSPNet in this study were 86.57% and 78.1%, respectively. The study in [24, 35] had 328 pterygium images, which are less than this study in terms of the number of training images. Simultaneously, the improved model can better extract image features and obtain better results.

Figure 6 shows that there is a certain gap between the segmentation and real results. The models can only assist physicians in determining the position before the surgery. Physicians also need to calibrate and confirm its boundary and range. More labeled data are required to further train the models, or a more sensitive and efficient model is expected. Therefore, the predicted segmentation results are closer to the real segmentation results.

## 5. Conclusions

A pterygium two-category model and a pterygium segmentation model for the images of the normal anterior and pterygium anterior segments were designed in this study, which could help patients self-screen easily and assist ophthalmologists in establishing the diagnosis of ophthalmic diseases and marking the actual scope of surgery. The VGG16 model can obtain the best diagnostic result among the four two-category models, and the double phase-fusion PSPNet model had the best results among the pterygium segmentation models. The two models could help patients self-screen easily and assist ophthalmologists in marking the actual scope of surgery.

## Figures and Tables

**Table 1:** Diagnostic results of the four models.

Clinical	AlexNet diagnosis		VGG diagnosis		ResNet18 diagnosis		ResNet50 diagnosis		Total
	Normal	Pterygium	Normal	Pterygium	Normal	Pterygium	Normal	Pterygium	
Normal	147	3	149	1	143	7	140	10	150
Pterygium	6	144	2	148	12	138	11	139	150
Total	153	147	151	149	155	145	151	149	300

**Table 2:** Evaluation index results of the four models.

Model	AlexNet		VGG16		ResNet18		ResNet50	
Evaluation indicators	Normal	Pterygium	Normal	Pterygium	Normal	Pterygium	Normal	Pterygium
Sensitivity	98.00%	96.00%	99.33%	98.67%	95.33%	92.00%	93.33%	92.67%
Specificity	96.00%	98.00%	98.67%	99.33%	92.00%	95.33%	92.67%	93.33%
F1-score	97.03%	96.97%	99.00%	99.00%	93.77%	93.56%	93.02%	92.98%
AUC	0.97		0.99		0.94		0.93	
95%CI	0.95–0.99		0.98–1		0.91–0.97		0.90–0.96	
Kappa	94.00%		98.00%		87.33%		86.00%	
Accuracy	97.00%		99.00%		93.67%		93.00%	

AUC: area under the curve; CI: confidence interval.

**Table 3:** Evaluation index results of the six models.

Model	MIOU (%)	IOU (%)	MPA (%)	PA (%)
U-Net	83.33	72.77	89.5	81.5
DeepLabv3+	83.91	73.98	91.45	86.39
PSPNet (MobileNet)	74.25	60.38	89.52	**88.89**
PSPNet (ResNet50)	85.4	76.27	91.92	86.7
**Phase-fusion PSPNet**	86.31	77.64	91.91	86.1
**Double phase-fusion PSPNet**	**86.57**	**78.1**	**92.3**	86.96

MIOU: mean intersection over union; IOU: intersection over union; MPA: mean average precision; PA: average precision.

## Data Availability

The data used in this study can obtain from the corresponding author with a reasonable request.
